# Influence of glucocorticoid treatment on trabecular bone score and bone remodeling regulators in early rheumatoid arthritis

**DOI:** 10.1186/s13075-021-02562-3

**Published:** 2021-07-06

**Authors:** Addolorata Corrado, Cinzia Rotondo, Angiola Mele, Daniela Cici, Nicola Maruotti, Eliana Sanpaolo, Ripalta Colia, Francesco Paolo Cantatore

**Affiliations:** Rheumatology Clinic, Department of Medical and Surgical Sciences, University of Foggia, Viale Pinto, 1, 71100 Foggia, Italy

**Keywords:** Rheumatoid arthritis, Glucocorticoids, Trabecular bone score, Bone mineral density, Wnt signaling

## Abstract

**Background:**

Glucocorticoids (GC) modulate several regulators involved in the pathogenesis of bone changes in rheumatoid arthritis (RA). Trabecular bone score (TBS) allows the indirect assessment of bone quality. The aim of this study was to investigate the effects of GC on TBS and serum levels of bone turnover regulators in patients with recent-onset RA.

**Materials and methods:**

Forty-seven subjects with recent-onset RA (< 6 months) were classified in two groups, low (lGC) and high (hGC) glucocorticoids, according to glucocorticoid dose regimens. Bone mineral density (BMD), TBS, and circulating Dickkopf-1 (Dkk1), sclerostin, osteoprotegerin (OPG), and RANK-L were evaluated at baseline and 6 and 12 months.

**Results:**

BMD significantly declined after 12 months with no significant difference between the lGC and hGC group, whereas TBS decreased in the hGC group only. Circulating OPG decreased during the follow-up period, the reduction being significantly greater in hGC group; conversely, sclerostin and RANK-L serum increased, in a significantly greater extent in the hGC group. TBS inversely correlated with sclerostin, RANK-L, and Dkk1 circulating levels whereas directly correlated with OPG circulating levels. GC cumulative dose showed an inverse relationship with BMD in both the hGC and lGC groups; TBS values showed an inverse relationship with GC cumulative dose in the hGC group only. GC cumulative dose was associated to higher sclerostin and lower OPG serum levels. TBS did not correlate with disease activity whereas BMD was inversely related to disease activity.

**Conclusions:**

In early RA, GC exposure contributes to the reduction of BMD and affects bone quality depending on dose regimens. TBS could be a useful tool to evaluate the negative effect of GC on bone microarchitecture.

**Trial registration:**

This study was ancillary to a parallel-group observational prospective study which was approved by the medical local ethics committee (protocol number DDG 334/19-06-2019).

## Background

Rheumatoid arthritis (RA) is a chronic systemic inflammatory disease mainly involving the synovial joints, and it is characterized by both local bone loss, represented by juxta-articular osteoporosis and erosions, and systemic bone loss, represented by generalized osteoporosis [1]. Generalized osteoporosis is more frequent in RA patients than in general population, and it is associated to an increased fracture risk. The physiopathology of osteoporosis in RA is very complex; particularly, systemic inflammation and glucocorticoid (GC) treatment are two major determinants of generalized bone loss; inflammatory cytokines, such as TNFα, IL6, and IL1 are associated to enhanced osteoclast activity, mainly mediated by receptor activator of nuclear factor kappa-Β ligand (RANKL), while GC-induced bone loss is related to both inhibition of bone formation mediated by the inhibitory effects on Wnt signaling and to an increase of bone resorption associated to the dysregulation of RANKL/osteoprotegerin (OPG) system [2].

The majority of patients with RA develop osteoporosis during the disease progression; nevertheless, there are few available data concerning bone changes in early RA. Dual X-ray absorptiometry (DXA) is the established standard for measuring bone mineral density (BMD), but it does not provide any information about the bone quality and bone microarchitecture, which are parameters very difficult to evaluate in clinical practice but essential to define bone strength [3]. Actually, BMD only partially represents bone strength, which results from both bone density and bone quality; indeed, in patients treated with GC, osteoporotic fractures occur with higher BMD values compared to untreated patients [4].

The trabecular bone score (TBS) is a new structural parameter that can be obtained from the textural greyscale analysis of DXA images. It is a structural index which allows the indirect assessment of bone microarchitecture, and it is able to provide data on bone quality irrespective of bone density [5]. It has been shown that TBS gives additional information concerning the alterations of bone quality associated to glucocorticoids treatment [6] and may be a valuable tool to evaluate bone quality changes independent of BMD.

The aims of this study were to investigate early changes of TBS in patients with recent-onset RA and to evaluate the relationship between bone changes and clinical variables, particularly glucocorticoids and disease activity, and serum levels of bone regulators of bone turnover.

## Patients and methods

The study included 47 subjects (36 females, 11 males) fulfilling the 2010 American College of Rheumatology/European League Against Rheumatism classification criteria for RA [7] and recent onset of joint symptoms (< 6 months of synovitis). Non-steroidal anti-inflammatory drugs and prednisone were allowed during the study time. Inclusion criteria were age > 18 years and body mass index (BMI) ranging from 15 to 35 kg/m^2^. Patients with metabolic disorders, secondary causes of osteoporosis, and post-menopausal women were excluded from the study, as well as patients with history of cancer, liver and kidney and endocrine diseases, inflammatory or rheumatic diseases other than RA, pregnancy or feeding time, diabetes mellitus, hyperparathyroidism, and hypercortisolism and obese patients (BMI > 30). Exclusion criteria included also patients or controls who had undergone a surgical procedure to spinal vertebrae or hips with thyroid or parathyroid disorders and use of calcium or drugs able to interfere with bone metabolism such as bisphosphonates for 6 months prior to the enrollment.

The recruited patients were treated according to the current EULAR recommendation for management of RA [8] and were divided into two groups based on GC dose regimens. The low glucocorticoid (lGC) group included patients receiving low dose glucocorticoid therapy (< 7.5 mg mean daily prednisone equivalent dose), while high glucocorticoid (hGC) group included patients treated with high glucocorticoid therapy (≥ 7.5 mg mean daily prednisone equivalent dose).

Demographic and clinical data, including disease-modifying anti-rheumatic drugs (DMARDs), GC treatment, and osteoporosis risk factors were evaluated at baseline and every 3 months for 12 months at each follow-up time point. All patients underwent physical examination and routine blood and urinary analysis at baseline and every 3 months for 12 months. The clinical activity of the disease was assessed at baseline and every 3 months until the end of the study by disease activity score (DAS28) evaluated with C-reactive protein.

The study protocol was approved by the local institutional ethics committee of University of Foggia – Ospedali Riuniti Foggia, and all subjects provided written informed consent before recruitment according to the Declaration of Helsinki.

### BMD and TBS evaluation

DXA measurements were performed in all participants using a total body scanner (QDR 4500 Acclaim Series Elite, Hologic Inc., Bedford, MA, USA) to evaluate BMD and T score. BMD was evaluated both at the lumbar spine (L1-L4) using the anteroposterior view and left hip (femoral neck and total hip) and was expressed as grams of bone mineral per square centimeters (g/cm^2^). All measurements were performed in accordance with the manufacturer’s standard instructions by the same operator and using the same DXA device. TBS was evaluated using the same DXA images of the lumbar spine (L1-L4), which were analyzed in an operator-independent manner with the TBS (iNight software version 2.1 Med-Imaps, Merignac, France). Both BMD and TBS were evaluated at recruitment and at 6 and 12 months of follow-up time points.

### Laboratory test

Measurements of the biologic serum variables were made on blood samples collected after an overnight fasting of 12 h at baseline, at 6 months, and at 12 months; the collected sera were frozen at − 20 °C until assay. Serum levels of sclerostin, dickkopf-1 (Dkk1), osteoprotegerin (OPG), and RANKL, all expressed as pg/ml, were measured using enzyme-linked immunosorbent assay (Human Dickkopf-1 ELISA Kit- Mybiosource, San Diego, US – mbs3802065; Human Osteoprotegerin ELISA Kit- Mybiosource, San Diego, US – mbs1758882; Human Sclerostin ELISA Kit – Thermo Fisher , Waltham, US - EHSOT; Human RANK-L ELISA Kit Mybiosource, San Diego, US mbs2702604).

### Statistical analysis

The results were expressed as mean ± SD or percentage. The normal distribution of data was assessed using the Shapiro–Wilk test. Changes observed at baseline and at the different time points of follow-up in each treatment group were assessed using ANOVA for repeated measures, followed by LSD test where appropriated. Correlation between continuous variables was assessed using the Pearson test. Factors associated with TBS and BMD were assessed by multivariate linear regression analysis. P value ≤ 0.05 was considered statistically significant. Statistical analysis was performed with IBM SPSS Statistic 23 software.

## Results

### Clinical and demographic characteristics

The main clinical and demographic characteristics of the enrolled subjects are illustrated in Table 1.

**Table 1:** Clinical and demographic characteristics at baseline

*Parameter*	
Sex, female, n (%)	38 (80.8%)
Age (years)	38.33 ± 7.03
DAS28	3.65 ± 0.71
Smokers, n (%)	11 (23.4%)
Alcohol	25 (53.1%)
ACPA, n (%)	44 (93.6%)
RF, n (%)	42 (89.36%)
BMI	23.64 ± 2.62
BMD spine (g/cm^2^)	0.877 ± 0.1
BMD femoral neck (g/cm^2^)	0.795 ± 0.1
BMD total hip (g/cm^2^)	0.854 ± 0.108
TBS	1.357 ± 0.401
Sclerostin (pg/ml)	285.6 ± 40.38
Dkk-1 (pg/ml)	3710.06 ± 772.7
OPG (pg/ml)	443.45 ± 80.57
RANKL (pg/ml)	277.74 ± 79.6
OPG/RANKL	1.83 ± 0.89

ACPA, anti-citrullinated protein antibody; RF, rheumatoid factor BMI, body mass index; BMD, bone mineral density; TBS, trabecular bone score; Dkk-1, Dickkopf; OPG, osteoprotegerin; RANKL, nuclear factor kappa-Β ligand

Forty-seven patients (36 women, 11 men), with a mean age of 38.33 ± 7.03, were included in the study. DAS28 at baseline was 3.65 ± 0.7. lGC consisted of 22 patients (16 F/6 M), treated with mean glucocorticoid daily dose of 4.27 ± 1.01 mg corresponding to a glucocorticoid cumulative dose of 1557.95 ± 445.73 mg during the follow-up time, whereas hGC consisted of 25 patients (20F/5M) treated with mean glucocorticoid daily dose of 9.46 ± 1.6 mg corresponding to a cumulative dose of 3454.67 ± 493.9. No differences in BMD, BMI, TBS, serum levels of OPG, RANKL, Dkk1, sclerostin, and demographic were observed between the hCG and lGC groups at baseline.

### Changes in BMD and TBS

**Fig. 1:**
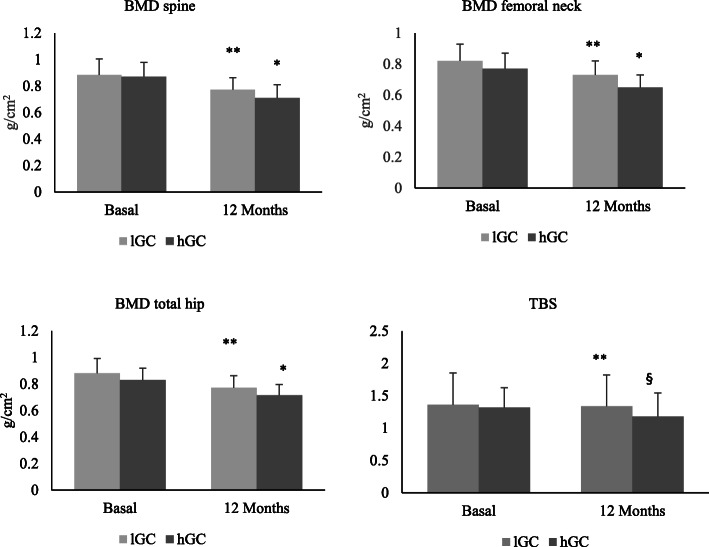
BMD and TBS values at baseline and after 12 months of follow-up in the lGC group and hGC group—in percent changes after 6 and 12 months of treatment with high and low GCs dose. Data are expressed as mean ± SD. **p < 0.05 vs baseline; *p < 0.005 vs baseline; ^§^p < 0.05 vs low GCs

A significant reduction of BMD at the spine, femoral neck, and total hip was observed after 12 months compared to baseline both in lGC (0.884 ± 0.09 vs 0.772 ± 0.79 at the spine; 0.820 ± 0.11 vs 0.735 ± 0.102 at the femoral neck; 0.882 ± 0.117 vs 0.788 ± 0.096 at the total hip) and in hGC group patients (0.871 ± 0.105 vs 0.740 ± 0.08 at the spine; 0.773 ± 0.09 vs 0.625 ± 0.08 at the femoral neck; 0.830 ± 0.09 vs 0.715 ± 0.083 at the total hip), with no significant difference between lGC group and hGC group (see Table 2) (Fig. 1).

**Table 2:** Comparison of baseline and 12 months characteristics between the low GC group and high GC group Results are presented as the mean ± SD

	Baseline		6 months		12 months		p value
	lGC (22)	hGC (25)	lGC (22)	hGC (25)	lGC (22)	hGC (25)	
Sex, female, n (%)	16 (72.7)	22 (88)					
Age (years)	37.3 ± 5.02	38.96 ± 7.7					
DAS28	3.6 ± 0.65	3.68 ± 0.77			2.85 ± 0.5**	3.02 ± 0.73*	*p < 0.05;**p < 0.05
Smokers, n (%)	5 (22.7)	6 (24)			4 (18.18)	6 (24)	
Alcohol	11 (50)	14 (56)	–	–	–	5 (20)	
ACPA, n (%)	20 (90.1)	24 (96)	–	–	–	–	
RF, n (%)	19 (86.3)	23 (92)	–	–	–	–	
Mean daily GC dose (mg)	–	–			4.27 ± 1.2	9.46 ± 1.62	
Cumulative GC dose (mg)	–	–			1557.95 ± 442.15	3454 ± 445.73	
BMI	23.27 ± 2.8	24.01 ± 2.6			23.05 ± 2.5	24.11 ± 2.45	
BMD spine (g/cm^2^)	0.88 ± 0.09	0.87 ± 0.1	0.83 ± 0.2	0.87 ± 0.1	0.77 ± 0.07**	0.71 ± 0.08*	*p < 0.0001; *p < 0.0001
BMD femoral neck (g/cm^2^)	0.82 ± 0.11	0.77 ± 0.09	0.82 ± 0.11	0.76 ± 0.09	0.73 ± 0.10*	0.65 ± 0.08*	*p < 0.0001; *p < 0.0001
BMD total hip (g/cm^2^)	0.88 ± 0.11	0.83 ± 0.09	0.87 ± 0.11	0.82 ± 0.09	0.78 ± 0.09**	0.71 ± 0.08*	*p < 0.0001; *p < 0.0001
TBS	1.363 ± 0.48	1.315 ± 0.31	1.352 ± 0.31	1.306 ± 0.329	1.34 ± 0.49	1.185 ± 0.37** §	*p < 0.0001;§p < 0.0001
OPG (pg/ml)	448.7 ± 17.3	438.8 ± 16.2	432.6 ± 16.1	411.4 ± 15.1	400.7 ± 18.7**	358.1 ± 17.7* §	*p < 0.0001; *p < 0.0001 §p < 0.0001
RANKL (pg/ml)	275 ± 17.1	280.1 ± 16.1	301.3 ± 95.8	441 ± 89.9	401.4 ± 26.5**	417.7 ± 24.9*	*p < 0.05; ** p < 0.05
Scl (pg/ml)	288.4 ± 8.6	283 ± 8.1	296.9 ± 9.5	314.4 ± 8.9	344.6 ± 15**	384.6 ± 14* §	*p < 0.0001; *p < 0.0001 §p < 0.0001
Dkk (pg/ml)	3736.1 ± 166.4	3688.2 ± 156.1	3781.2 ± 147.2	3710.4 ± 138.1	3750 ± 142.3	3726.2 ± 133.4	0.773

**12 months vs baseline in the low GC group; *12 months vs baseline in the high GC group; § high vs low GC

After 12 months follow-up, no significant changes in TBS values were observed in lGC group patients; conversely, hGC group patients showed a significant reduction of TBS score compared to baseline (1.35 ± 0.31 vs 1.185 ± 0.37), thus presenting significantly lower TBS values compared to patients treated with low glucocorticoid doses (1.340 ± 0.49 vs 1.185 ± 0.37) despite similar BMD (see Table 2).

No correlation was found between TBS and BMD at the spine, total hip, and femoral neck, both at baseline then after 12 months of follow-up.

None of the patients recruited experienced fractures during the follow-up period.

### Changes in OPG/RANKL, Dkk1, and sclerostin

**Fig. 2:**
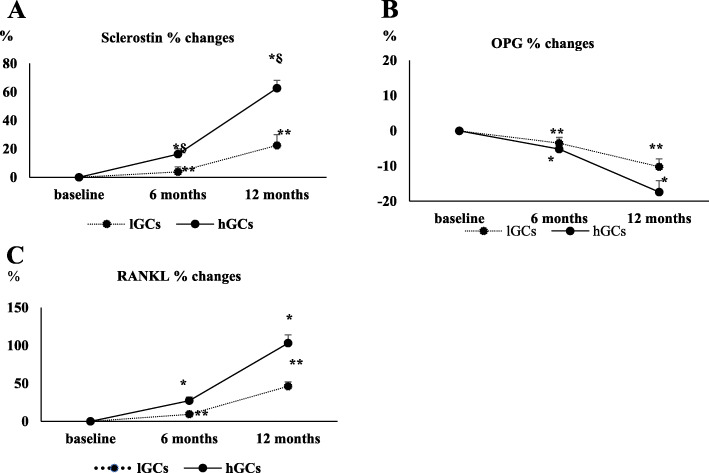
Sclerostin, OPG, and RANKL percent changes after 6 and 12 months of treatment with high and low GCs dose. Data are expressed as mean ± SE. * p < 0.05 vs baseline; ^§^p < 0.05 vs low GCs

OPG serum levels significantly decreased after 6 months and 12 months of follow-up both in the lGC group (− 3,6% and − 10.7% respectively ) and in the hGC group (− 6.2% and − 18.1% respectively) (p < 0.001); the reduction of OPG levels was significantly greater in the hGC group in comparison to the lGC group (p < 0.05) (Table 2). Concerning RANKL serum concentration, it significantly increased after 12 months, both in the lGC group (+ 9.56% and + 45.9% respectively, p < 0.04) and in the hGC group (+ 57.4% and + 49.1%, respectively), with no differences at 6 and 12 months between the two groups. A significant increment of sclerostin serum levels was observed at 12 month both in lGC group (+ 2.94% and + 19.48%), respectively, and in the hGC group (+ 11.1% and + 35.88% respectively); in the hGC group, the serum sclerostin levels were significantly higher in comparison with lGC group (384.65 ± 70.58 pg/ml vs 344.6 ± 70.58 pg/ml at 12 months). No changes in Dkk1 circulating levels were observed in both glucocorticoid treatment groups at the end of the study (Fig. 2; see Table 2).

### Relationship between TBS, Wnt signaling antagonists, and OPG/RANKL

At baseline, in the whole group of patients, univariate linear regression analysis showed that TBS was negatively associated to activity disease, expressed ad DAS28 (DAS28) (β = − 0.31; p = 0.034) and to RANK-L (β = − 0.655; p = 0.0001); conversely, a positive association with OPG was found (β = 0.446; p = 0.002). Multiple linear regression showed a negative association with DAS (β = − 0.261; p = 0.043), RANKL (β = − 0.425; p = 0.001) and DKK (β = − 0.572) levels.

After 12 months, by univariate analysis on the whole group of patients, the significant association of TBS with activity disease was not observed and no correlation was found between TBS and disease activity; conversely, TBS showed a negative association with Scl (β – 0.45; p = 0.001), RANKL (β – 0.714, p = 0.0001), and DKK (β – 0.83, p = 0.0001) serum levels and a positive association with OPG (β 0.57; p = 0.0001). Multivariate regression analysis confirmed the negative association with DKK (β – 0.549, p = 0.001) and the positive association with OPG (β 0.25, p = 0,007). Further, TBS values showed a significant negative correlation with serum levels of sclerostin (r^2^ − 0.43, p < 0.003), RANKL (r^2^ − 0.76, p < 0.001), Dkk-1 (r^2^ − 0.86, p < 0.001), and a significant positive correlation with OPG (r^2^ 0.43, p < 0.002).

In the whole group of patients, the cumulative dose of GC was not a predictive factor for TBS and no correlation between TBS and the cumulative glucocorticoid dose was observed; nevertheless, in the hGC group, TBS showed a significant negative correlation with the cumulative dose of glucocorticoids (r^2^ -− 0.39, p < 0.04); in the hCG group, no correlation with disease activity was observed, as well as in the whole group patients.

At 12 months of follow-up, the cumulative dose of CG was a negative predictive factor associated to the spine BMD (β – 0.36, p = 0.013) and total hip BMD (β – 0.397, p = 0,007); univariate analysis confirmed the negative predictive value of cumulative GC dose on total hip BMD (β – 0.304, p = 0,039) and femoral neck BMD (β − 0,348, p = 0,018). Further, BMD showed an inverse relationship with the cumulative glucocorticoid dose at the spine (r^2^ − 0.86, p < 0.042), femoral neck (r^2^ − 0.41, p < 0.04), and total hip (r^2^ − 0.41 p < 0.04). Disease activity was not a predictive factor of BMD; nevertheless, an inverse relationship between disease activity and BMD at the spine, femoral neck, and total hip was found (r^2^ − 0.30, p < 0.04; r^2^ − 0.30; p < 0.04; r^2^ − 0.40 p < 0.04 respectively).

The cumulative glucocorticoid dose showed a positive correlation with serum level of sclerostin and a negative correlation with serum levels of OPG (r^2^ − 0.32, p = 0.02; r^2^ − 0.378, p = 0.009).

No correlation between disease activity and serum level of OPG, RANKL, sclerostin, and Dkk1 was found. Correlation between TBSm cumulative dose of GC and serum bone markers are shown in Table 3.

**Table 3:** Correlation between TBS and GC cumulative dose with different serum bone markers in the whole group of patients, in the lGC group and hGC group

	All patients (N = 47)		Low GC group (N = 22)		High GC group	
*Variable*	*TBS*	*Cumulative GC dose*	*TBS*	*Cumulative GC dose*	*TBS*	*Cumulative GC dose*
TBS	–		–		–	
Cumulative dose GC	− 0.19 (ns)	–	0.003 (ns)	–	**− 0**.**4 (p = 0**.**048)**	–
DKK	**− 0**.**86 (p < 0**.**0001)**	0.12(ns)	**− 0**.**82 (p < 0**.**0001)**	0.62	**− 0**.**9 (p < 0**.**0001**)	0.42 (p = 0.033)
Sclerostin	**− 0**.**43 (p = 0**.**003)**	**0**.**32 (p = 0**.**026)**	**− 0**.**58 (p = 0.004)**	0**.**07 (ns)	**− 0.47 (p = 0.04)**	0.16 (ns)
RANK-L	**− 0.76 (p < 0.001)**	0**.**14 (ns)	**− 0.75 (p < 0.0001)**	0**.**54 (ns)	**− 0.71 (p < 0.0001)**	0.22 (ns)
OPG	**0.43 (p = 0.002)**	**− 0.37 (p = 0.009)**	0**.**38 (ns)	**−** 0**.**9 (ns)	**0.45 (p = 0.023)**	**−** 0.33 (ns)
OPG/RANK-L	**0.76 (p < 0.0001)**	**−** 0**.**23 (ns)	**0.78 (p < 0.0001)**	**−** 0**.**11 (ns	**0.74 (p < 0.0001)**	**−** 0.32 (ns)

## Discussion

GC have a fundamental role in the treatment of RA [9–12]; despite their detrimental effects on BMD being well known [13–16], GC can contribute to reduce local and systemic bone loss by the anti-inflammatory effect that may counteract the negative effect on bone [17–19]. It has been shown that systemic bone loss occurs very early in RA [20], with a high rate of reduction of BMD in patients with few weeks of disease duration and not treated with GC and a significant reduction of BMD appearing after 1 year of GC treatment [21]; conversely, a significant BMD decrease after the first month of GC treatment [22] has been observed; to date, there are very few data concerning the effects of GC on bone in early RA. BMD obtained by DXA is the reference parameter for the evaluation of bone loss [23], but it is not the appropriate tool to evaluate bone quality. TBS is obtained by greyscale textural analysis of DXA scans of the lumbar spine and reflects bone microarchitecture, providing additional information that cannot be obtained by standard BMD assessment. Higher TBS correlate with better bone microarchitecture, while low TBS is related to poorer bone microarchitecture in spite of identical BMD [24]. It has been shown that patients receiving GC treatment for systemic inflammatory diseases presented a significant decrease of TBS, in greater extent compared to the reduction of BMD [25].

The presented data showed a significant decline of BMD in early RA both at the spine and hip after 12 months from disease onset, whereas a significant reduction of TBS was observed only in patients receiving high GC doses. Interestingly, there are no significant differences in spine and hip BMD between patients treated with high and low cumulative GC dose, whereas TBS values at 12 months were significantly lower in patients receiving the greatest cumulative dose of GC.

These findings are consistent with the notion that in the early stage of disease, GC treatment is related to bone microarchitecture changes irrespective of BMD changes [25]. A previously published study showed that women treated with GC presented lower TBS values compared to untreated women, whereas the BMD values did not differ between the two groups [6].

One might hypothesize that in the initial stage of disease, high GC doses can contribute to the deterioration of bone strength by early alteration of bone quality, in addition to the reduction of BMD. Disease activity is a factor that can affect both BMD and TBS; nevertheless, in the presented report, disease activity was a negative predictive factor of TBS only at baseline, whereas after 12 months, disease activity was not associated to TBS; conversely, disease activity was not a predictive factor of reduced BMD (both at baseline and at 12 months), although BMD showed an inverse relationship with disease activity. This could be explained by the fact that even if disease activity does not directly affect the BMD values, patients with higher activity of disease may require higher doses of GC, which are a negative predictive factor of BMD values. On the other hand, concerning TBS, these results could lead to hypothesized that in the early stage of disease, in untreated patients, disease activity affect bone microarchitecture; in the following phases of disease, after the begin of treatment, other factor may be related to changes in bone architecture, including treatment with GC.

An essential aspect of the pathogenesis of GC-induced bone changes is the suppression of bone formation [26]; the inhibition of Wnt/β-catenin signaling is one of the mechanisms by which GC reduce osteoblast function [27]. In vitro studies showed that GC induce an impairment of osteoblast maturation and function through a dose-dependent suppression of the canonical Wnt/β-catenin pathway and an increased expression of Wnt antagonists, such as Dkk1 and sclerostin [28, 29], as shown in experimental animal models [30–32].

In vivo human studies evaluating the effects of GC on Wnt signaling pathway are not consistent. While reduced circulating sclerostin has been shown in the first week of GC treatment [33], serum sclerostin and Dkk-1 levels have been found to significantly increase after 1 week of GC treatment and decrease afterwards [32]; recently, a reduction of Dkk-1 and sclerostin levels has been shown in early RA patients after 4 weeks of GC treatment [34]. Other studies report increased sclerostin levels after a longer period of GC treatment [35] and in long-term supraphysiological levels of GC due to Cushing’s syndrome [36].

These conflicting results may be due to the different clinical conditions and can vary depending on therapy duration and dose, the inflammatory status, and the different underlying disease.

In this study, we found that sclerostin serum levels increased after 12-month follow-up to a significant greater extent in patients treated with higher GC doses; further, serum levels of sclerostin and DKK were negatively associated to TBS and a positive correlation between serum sclerostin levels and cumulative GC dose was observed. On the other hand, no significant correlation between GC use and Dkk1 was found. In other studies, reduced Dkk1 serum levels have been described in patients with hematological disorders [35] and in post-menopausal women [37] treated with high GC. Despite being conflicting, these results suggest that GC could act on bone metabolism by modulating Wnt signaling.

GC contribute to bone deterioration through other mechanisms, including increased osteoclast activity. GC enhance the expression of macrophage colony stimulating factor and RANKL, while reducing the expression of its soluble decoy receptor, OPG, in stromal and osteoblastic cells [32], determining, at least in an early stage, an increased osteoclast activity. OPG/RANKL system plays a key role in the pathogenesis of systemic and local bone loss in RA being associated to disease progression [38–41] and is affected by several factors other than GC treatment, including inflammation status and activity disease [42].

After 12 months of follow-up, we found that RANKL serum levels were significantly increased both in the lGC group and in the hGC group, and circulating RANKL levels inversely correlated with TBS values; conversely, OPG significantly declined compared to baseline, showing a significant greater reduction in patients treated with higher GC dose. Further, RANKL resulted as a negative predictor of TBS, whereas OPG was positively associated with TBS and a positive relationship between OPG serum levels and TBS was found.

No correlation between both OPG and RANKL serum levels and disease activity was found; thus, it could be supposed that the effects of GC on RANKL/OPG system and on Wnt regulators, which in the early stage of disease do not appear to be related to disease activity, could account for the deterioration of bone quality, expressed as TBS, particularly in patients receiving high glucocorticoid dose treatment.

## Conclusions

Data on TBS in the early stage of RA are at present very limited. The findings of this study show that in the early stage of RA, GC and disease activity contribute to reduction of BMD and suggest that GC exposure has a significant impact on bone microarchitecture mediated by the suppression of Wnt signaling and the modulation of OPG/RANKL system. Further, these data underline the potential role of TBS in detecting the early changes of bone microarchitecture, suggesting that TBS could provide supplementary information in addition to BMD to evaluate bone status in RA. The main limitation of this study is represented by the relatively small number of recruited subjects; it is actually a limited case-series, but it should be considered that the inclusion criteria are very stringent and patients have been recruited in a single center. Further investigations are required to evaluate the relationship between GC treatment and TBS changes and the possible role of other factors, including DMARDs, which could affect TBS.

## Data Availability

The datasets used and/or analyzed during the current study are available from the corresponding author on reasonable request.

## Abbreviations

ACPA: Anti-citrullinated protein antibody
BMD: Bone mineral density
BMI: Body mass index
DAS28: Disease activity score
Dkk1: Dickkopf-1
DMARDs: Disease modifying anti-rheumatic drugs
DXA: Dual X-ray absorptiometry
GC: Glucocorticoids
hGC: High glucocorticoids
lGC: Low glucocorticoids
OPG: Osteoprotegerin
RA: Rheumatoid arthritis
RF: Rheumatoid factor
RANKL: Receptor activator of nuclear factor kappa-Β ligand
TBS: Trabecular bone score
